# Using Local Data on Adults Aged 18 to 64 to Tailor Interventions for Blood Pressure Medication Adherence in Maine

**DOI:** 10.5888/pcd16.180456

**Published:** 2019-06-20

**Authors:** Caitlin Pizzonia, David Pied, Sara L. Huston, Pamela Foster Albert, Gregory Parent, Nathan Morse

**Affiliations:** 1 Cutler Institute for Health and Social Policy, Muskie School of Public Service, University of Southern Maine, Portland, Maine; 2 Chronic Disease Program, Division of Disease Prevention, Maine Center for Disease Control and Prevention, Augusta, Maine

**Figure:**
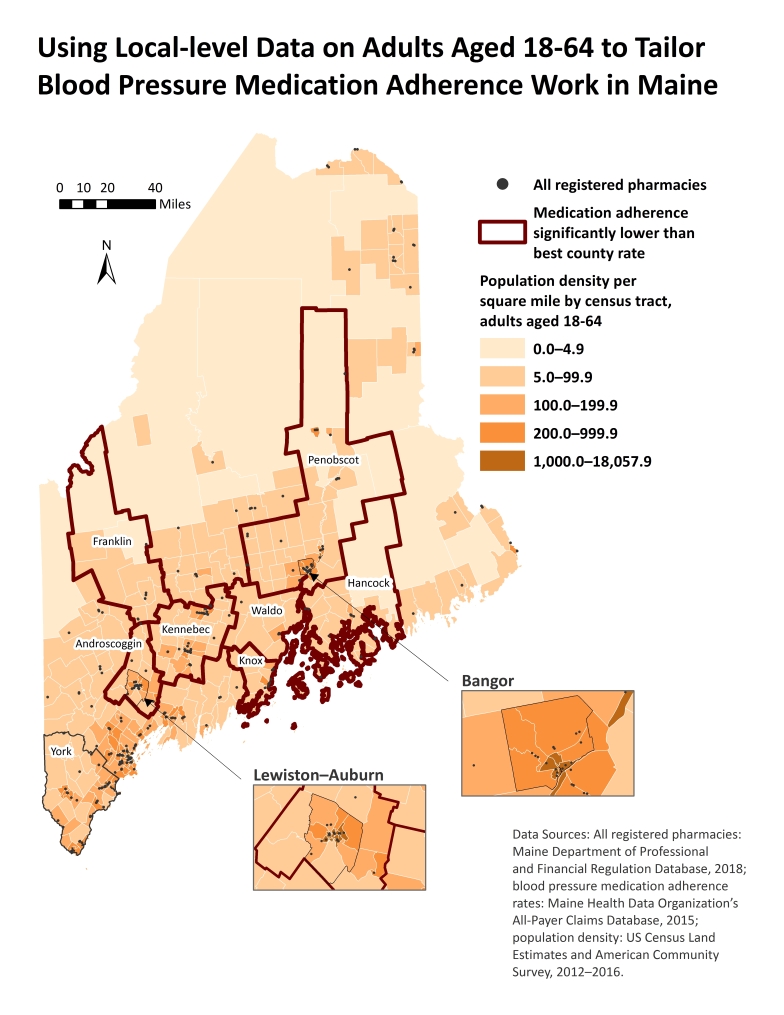
Maine licensed pharmacy locations, blood pressure medication adherence rates, and population density in 2018. Medication adherence in 2015 among Maine adults aged 18 to 64, calculated for renin–angiotensin system antagonists by using the proportion-of-days-covered method, was 83.8% (95% confidence interval, 83.4%­–84.1%). York County had the highest adherence rate (85.2%; 95% confidence interval, 84.3%–86.0%). Counties with medication adherence rates significantly lower than the York County rate indicate where to focus interventions. Adult census tract–level population density for 2012 through 2016 indicates where to implement rural-specific interventions.

## Background

Maps showing US state-level data help researchers understand the geographic distribution of chronic disease burden. As public health analysts refine spatial analysis skills, sub-state analyses are sought to determine how to tailor interventions to specific populations so that public health programs can use limited funds most effectively. A workgroup at the Division of Disease Prevention at the Maine Center for Disease Control and Prevention (Maine CDC) created this map to determine where and how to administer public health programs among adults aged 18 to 64 to increase adherence to antihypertensive medication regimens, ultimately influencing hypertension control rates.

In 2015, one in 3 Maine adults (33.4%) had diagnosed hypertension (1), and in 2015–2016 only half of Americans with diagnosed hypertension had controlled hypertension (2). Adherence to antihypertensive medication is associated with controlled hypertension and reduced risk of cardiovascular events (2). US costs for hypertension without heart disease, including health care services, medications, and missed work, totaled $55.9 billion in 2014–2015 dollars (3). Recent evidence also shows that adults aged 35 to 64 are less likely than adults aged 65 or older to take blood pressure medication and have controlled hypertension, thereby increasing their risk for heart disease and stroke (4). Reducing hypertension and cardiovascular events are public health priorities and Healthy People 2020 indicators (5). The Million Hearts program, a national initiative to prevent 1 million heart attacks and strokes, promotes efforts to control hypertension through increasing medication adherence and self-measured blood pressure monitoring (6).

Because half of Maine’s land area is uninhabited and 40.8% of the state population lives in rural counties, population density is a critical component in understanding rural health needs. Rural areas tend to have more veterans, older adults (≥65 y), and residents living in poverty than urban areas (7), and rural residents may face unique challenges in controlling hypertension, such as living long distances from pharmacies or physicians.

## Date Sources and Map Logistics

Licensed pharmacy locations were received from the 2018 Maine Department of Professional and Financial Regulation Database and geocoded (8). Adult population density, representing adults aged 18 to 64, was calculated from the 2012–2016 US Census population estimates by dividing the total population of adults aged 18 to 64 by the census tract land area (square miles) (9,10). Five manual breaks were used to show variation in adult population density.

Pharmacy claims from the 2015 Maine Health Data Organization’s All-Payer Claims Database (APCD), used to calculate blood pressure medication adherence rates, represent claims from private and Medicaid beneficiaries (11). Though some private beneficiaries aged 65 to 85 may be Medicare Part D beneficiaries, not all Medicare Part D claims are included in the APCD. Medication adherence was calculated according to Centers for Disease Control and Prevention (CDC) 1305 grant guidance, which uses the proportion-of-days-covered method (having medication for ≥80% of total enrollment days) (12).

Because pharmacy claims could not be linked to medical claims in the Maine APCD, we limited our analysis to renin–angiotensin system antagonists (RASAs), which are used exclusively for hypertension (RASA medications include angiotensin converting enzyme inhibitors, angiotensin II receptor blockers, and direct renin inhibitors and are used only for hypertension, unlike other antihypertensive medications). Total enrollment days were calculated from the patient’s prescription start date through December 31, 2015 (12). Antihypertensive medication adherence was calculated among Maine adults aged 18 to 85 who filed 2 or more prescription claims for RASAs and had medication for at least 90 continuous days in 2015. Medication adherence rates were calculated among 2 age groups, 18 to 64 and 65 to 85, and Pearson χ^2^ tests (*P* < .05) were used to determine significance between the 2 age groups. County rates among adults aged 18 to 64 were analyzed, and a Pearson χ^2^ test (*P* < .05) was used to compare county-specific medication adherence rates to the Maine rate. Then, significance was determined by using a one-way ANOVA and least significant differences test (*P* < .05), comparing the best county rate to all other counties.

SAS version 9.4 (SAS Institute Inc) was used to calculate medication adherence rates and perform statistical analyses. No institutional review board approval was required, but we completed a data use agreement with the Maine Health Data Organization. The map was produced in ArcGIS version 10.6 (Esri).

## Highlights

Antihypertensive medication adherence rates increased with age and were significantly lower among adults aged 18 to 64 (83.8%; 95% confidence interval [CI], 83.4%–84.1%) than among adults aged 65 to 85 (86.9%; 95% CI, 86.6%–87.1%). Adherence rates among adults aged 18 to 64 were significantly higher in York County (85.2%; 95% CI, 84.3%–86.0%) than in Maine overall (83.8%; 95% CI, 83.4%–84.1%). Androscoggin, Franklin, Hancock, Kennebec, Knox, Penobscot, and Waldo counties had significantly lower medication adherence rates (*P* < .05) than York County, indicating where to tailor interventions (Table).

**Table:** Medication Adherence Among Adults Prescribed RASA Medications, by Demographic and Geographic Characteristics, Maine, 2015^a^

Characteristic	No. (%)^b^	% Adherent^c^ (95% CI)	*P* Value^d^
**Total**	105,663 (100)	85.5 (85.3–85.7)	NA
**Age, y**			
18–64	45,544 (43.1)	83.8 (83.4–84.1)	<.001
65–85	60,119 (56.9)	86.9 (86.6–87.1)	
**County, adults aged 18–64 y**			
Androscoggin	3,944 (8.7)	82.8 (81.6–84.0)	<.05^e^
Franklin	849 (1.9)	80.9 (78.3–83.6)	
Hancock	1,581 (3.5)	82.2 (80.3–84.1)	
Kennebec	4,366 (9.6)	82.8 (81.7–83.9)	
Knox	1,142 (2.5)	81.6 (79.4–83.9)	
Penobscot	5,829 (12.8)	83.4 (82.5–84.4)	
Waldo	1,383 (3.0)	80.2 (78.1–82.3)	
York	6,573 (14.4)	85.2 (84.3–86.0)	—f

Abbreviations: CI, confidence interval; NA, not applicable; RASA, renin–angiotensin system antagonists.

a From Maine Health Data Organization, All Payer Claims Database (APCD) (11).

b Maine adults aged 18–85 recorded in APCD pharmacy claims who filled at least 2 prescriptions for RASA (renin–angiotensin system antagonists) medications in 2015 that totaled at least 90 continuous days’ supply, with the first prescription filled on or before September 30, 2015. Study population numbers may not sum to group totals because of missing information on that demographic or geographic characteristic, and study population percentages may exceed 100% because of rounding.

c Percentage of Maine adults aged 18 to 85 adherent to medication regimens (had medication for 80% of days from the patient’s prescription start date until the end of the calendar year) based on 2015 Maine Department of Health APCD pharmacy claims.

d *P* values were calculated based on Pearson χ^2^ test at α = 0.05 between demographic groups.

e *P* values calculated by using the ANOVA test at α = 0.05 comparing York County to all Maine counties. Only significant counties were presented in this table.

f No *P* value was presented for York County because it is the county comparison rate.

Population density and pharmacy locations were concentrated in southern coastal regions but varied substantially within counties with lower medication adherence. Inset maps of Lewiston–Auburn (Androscoggin County) and Bangor (Penobscot County) were included in the map accompanying this article to show greater detail for population-dense towns in counties with lower medication adherence rates. A limitation of the map is that medication adherence rates may be slightly overestimated because of study inclusion criteria, the proportion-of-days-covered method, and restricting analyses to RASAs only. The map does not display medication adherence rates and is best used alongside data tables or an internal interactive ArcGIS online web application that the Division of Disease Prevention workgroup created.

## Action

Maine CDC could use data presented in the map to focus future tailored interventions in pharmacies located in counties with significantly lower adherence rates and replicate successful practices in York County pharmacies to improve medication adherence. Maine CDC can help implement self-measured blood pressure monitoring, lifestyle change programs, or telehealth interventions on the basis of local population density. Self-measured blood pressure monitoring and telehealth may be beneficial in areas with low population density because in-person lifestyle change programs may be less effective if patients live far away from a retail pharmacy. Applying all 3 approaches in densely populated areas could improve medication adherence and hypertension control for a high proportion of the state’s younger adult population (18–64 y). If the interventions increased medication adherence in identified lower adherence counties, 495 adults aged 18 to 64 taking RASA medications would be adherent to blood pressure medications, increasing their chances of controlled hypertension. This conservative estimate was calculated by multiplying the difference between the county rate and the York County rate (85.2%) by the county-level study populations (Table) and summing the results. The Maine CDC plans to replicate this collaborative map process to strategically inform other chronic disease prevention programs.
